# Sex differences in the risk of arterial stiffness among adults with different glycemic status and modifications by age

**DOI:** 10.1111/1753-0407.13353

**Published:** 2023-01-17

**Authors:** Xiaoyun Zhang, Qianqian Yang, Ruizhi Zheng, Zhiyun Zhao, Mian Li, Tiange Wang, Min Xu, Jieli Lu, Shuangyuan Wang, Hong Lin, Weiqing Wang, Guang Ning, Yufang Bi, Yu Xu, Yuhong Chen

**Affiliations:** ^1^ Department of Endocrine and Metabolic Diseases Shanghai Institute of Endocrine and Metabolic Diseases, Ruijin Hospital, Shanghai Jiaotong University School of Medicine Shanghai China; ^2^ Shanghai National Clinical Research Center for Metabolic Diseases, Key Laboratory for Endocrine and Metabolic Diseases of the National Health Commission of the PR China Shanghai Key Laboratory for Endocrine Tumor, State Key Laboratory of Medical Genomics, Ruijin Hospital, Shanghai Jiaotong University School of Medicine Shanghai China

**Keywords:** age, arterial stiffness, glycemic status, sex difference, 性别差异, 动脉硬化指数, 血糖状态, 年龄

## Abstract

**Background:**

Studies indicate lower, comparable, and higher cardiovascular risks in women vs men in normal glucose regulation (NGR), prediabetes, and diabetes, respectively. However, this sex difference is uncertain and aging might play a part. We aimed to estimate sex differences in arterial stiffness in NGR, prediabetes, or diabetes and the potential modifications by age.

**Methods:**

We used baseline data of 9618 participants aged ≥40 years in a large community‐based cohort study in Shanghai. Glycemic status was determined by history of diabetes, fasting and 2‐h post‐load glucose levels, and hemoglobin A1c levels. Arterial stiffness was examined by brachial‐ankle pulse wave velocity (ba‐PWV). Multivariable linear regression analysis was conducted to examine the associations between sex and ba‐PWV levels in glycemic and age categories.

**Results:**

Before adjustment for age, women had lower, comparable, and higher ba‐PWV vs men in the NGR, prediabetes, and diabetes groups, respectively. In participants aged 40–59 years, women were associated with lower ba‐PWV levels in generally all glycemic strata after adjustment for age and other confounders. In participants aged ≥60 years, women were associated with significantly higher ba‐PWV levels (*β* coefficient = 71.5; 95% confidence interval = 23.4, 119.7) and the sex difference was attenuated in the groups of prediabetes and diabetes with a borderline significant interaction between sex and glycemic status (*p* for interaction = .068).

**Conclusions:**

The sex difference in cardiovascular risks in adults with NGR, prediabetes, or diabetes was dependent on age. Our findings provide new evidence for prioritizing preventive treatment against atherosclerosis in men vs women with different glycemic status.

## Graphical abstract

**Highlights**

- Sex difference regarding arterial stiffness in different glycemic status and whether aging plays a role remains uncertain.
- In participants aged 40–59 years, women were associated with lower ba‐PWV levels in generally all glycemic strata after multivariable adjustment. In participants aged ≥60 years, women were associated with significantly higher ba‐PWV levels in the group of normal glycemic regulation after multivariable adjustment and this sex difference was attenuated in the groups of prediabetes and diabetes.
- Our findings provided additional evidence for targeted sex‐specific prevention of atherosclerosis in participants with different glycemic status.

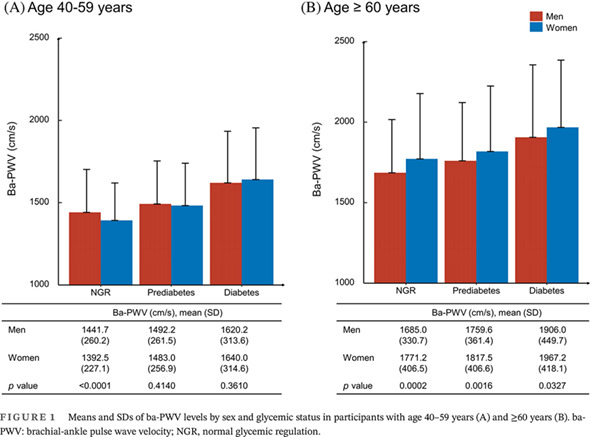

## 摘要

**背景:**研究表明在正常血糖调节(NGR)、糖尿病前期和糖尿病患者中, 女性的心血管风险分别较低、相当和较高。然而, 这种性别差异是不确定的, 年龄可能是原因之一。我们旨在评估NGR、糖尿病前期或糖尿病患者动脉硬化的性别差异, 以及年龄对动脉硬化的潜在影响。

**方法:**我们使用了上海一项大型社区队列研究中9618名≥40岁参与者的基线数据。血糖状态由糖尿病史、空腹和餐后2h血糖水平以及糖化血红蛋白水平决定。肱‐踝动脉脉搏波传导速度(ba‐PWV)检测动脉硬化水平。采用多变量线性回归分析检验性别、血糖、年龄与ba‐PWV之间的关系。

**结果:**在校正年龄前, 在NGR组、糖尿病前期组和糖尿病组中, 女性的ba‐PWV分别比男性低、相当和高。在40‐59岁的参与者中, 在校正年龄和其他混杂因素后, 女性在所有血糖水平分类中的ba‐PWV水平都较低。在≥60岁的参与者中, 女性的ba‐PWV水平显著升高(β=71.5, 95%可信区间=23.4,119.7), 性别差异在糖尿病前期和糖尿病组中减弱, 性别和血糖状态之间存在接近显著的交互作用(P_相互_=0.068)。

**结论:**在患有NGR、前驱糖尿病或糖尿病的成年人中, 心血管风险的性别差异取决于年龄。我们的发现为优先预防不同血糖状态的男性和女性动脉粥样硬化提供了新的证据。

## 1 INTRODUCTION

Cardiovascular disease (CVD), traditionally considered a common disease of men, now has become the leading cause of death in women, which accounts for more than one third of all deaths in the gender.^1^ However, CVD in women remains understudied, underrecognized, underdiagnosed, and undertreated.^2^

It is widely known that men have an excess risk of CVDs than women. A 25% higher risk in men vs women was found by the Prospective Urban and Rural Epidemiological (PURE) study.^3^ However, this advantage in women seems to be attenuated with the deterioration of glycemic regulation. In contrast to a significantly increased risk in the general population in men compared with women, findings from previous studies in people with prediabetes were inconsistent in the sex‐specific risk of CVD, which was reported higher,^4^, ^5^ similar,^6^, ^7^ or lower^8^, ^9^ in women compared with men. In people diagnosed with type 2 diabetes, emerging evidence revealed that women might have a higher risk of CVD compared with men.^10^ However, inconsistent results were also found.^11^, ^12^ Although the sex‐specific CVD risk remains uncertain, prevalence of cardiometabolic diseases in men and in women is highly dependent on age. Women tend to have lower prevalence than men before age 60, whereas after age 60, cardiometabolic diseases are likely to be more prevalent in women than in men.^13^, ^14^, ^15^

Subclinical atherosclerosis such as arterial stiffness is an early stage of CVD before manifestation of clinical symptoms and onset of cardiovascular events. Arterial stiffness measured using the pulse wave velocity (PWV) is reported to play a critical role in the process of vascular aging, promoting the initiation and progression of CVD.^16^ Brachial‐ankle pulse wave velocity (ba‐PWV) is often used to measure arterial stiffness in epidemiological studies given its high reproducibility and low cost.^17^, ^18^ Sex, age, and glucose dysregulation all have great impacts on arterial stiffness but findings were inconsistent.^19^, ^20^, ^21^ Therefore, using cross‐sectional data from a large community cohort in Shanghai, China, we aimed to elucidate the sex difference in the risk of arterial stiffness among adults with different glycemic status and to examine the potential modification effects by age.

## 2 MATERIALS AND METHODS

### 2.1 Study population

Participants aged over 40 years were enrolled from an ongoing community‐based cohort with baseline examination conducted between March 2010 and August 2010 in the Jiading District of Shanghai, China. The detailed information on study design has been well illustrated elsewhere.^22^ Briefly, a total of 10 375 participants were invited for a comprehensive examination of cardiometabolic health using face‐to‐face questionnaire interviews, physical examinations, and laboratory measurements. In the current analysis, we excluded participants with self‐reported CVD history (*n* = 304), and participants with missing data on ba‐PWV measurements (*n* = 420) or glucose measurements (*n* = 33). Finally, 9618 participants were included in the current analysis.

The study protocol was approved by the Institutional Review Board of Ruijin Hospital, Shanghai Jiaotong University School of Medicine ((2011) No.14, approved on 10 March 2011). The study complied with the guidelines of the Declaration of Helsinki, and written informed consent was acquired from each participant before study.

### 2.2 Data collection

A standard questionnaire was used by trained staff to collect information on demographic characteristics, socioeconomic status, lifestyle factors, disease history and medication use from each participant through a face‐to‐face interview. Education attainment was self‐reported and categorized into high school or above vs less than high school. Participants with self‐reported habits of smoking cigarettes or consuming alcohol regularly within the past 6 months were identified as current smokers or current drinkers, respectively. Physical activity was recorded using the short form of International Physical Activity Questionnaire with questions on the intensity, frequency and duration of physical activity.^23^ Being physically active was defined as ≥75 min per week vigorous‐intensity or ≥150 min per week moderate‐intensity or ≥150 min per week moderate‐plus vigorous‐intensity of physical activity according to the recommendations of the American Heart Association.^24^

Anthropometric and blood pressure measurements, blood sampling and biochemical tests were performed according to standardized procedures.^25^ Body height and body weight were measured while participants were dressed in light‐weight clothes and not wearing shoes. Body mass index (BMI) was calculated as body weight in kilograms divided by body height in meters squared (kg/m^2^). Three consecutive blood pressure measurements were conducted with 1‐min intervals between measurements after at least a 5‐min rest in the seated position using a calibrated automated electronic device (OMRON Model HEM‐752 FUZZY; Omron Company, Dalian, China) and participants were asked to avoid exercise, cigarettes, alcohol, coffee, tea, or medicine for at least half hour before measurement. We used the average of three readings in the current study. Venous blood samples were drawn from each participant after at least 10 h of overnight fasting. Participants without a history of diabetes underwent a standard oral glucose tolerance test (OGTT) and blood was drawn at 0 and 2 h during the test. Biochemical parameters including plasma glucose, low‐density lipoprotein cholesterol, high‐density lipoprotein cholesterol (HDL‐c), triglycerides, serum creatinine, and serum insulin were determined by the auto‐analyzers (Modular Analytics P800 and Modular E170; Roche, Basel, Switzerland). Hemoglobin A1c (HbA1c) was determined by high‐performance liquid chromatography (Bio‐Rad Laboratories, Hercules, CA, USA). The homeostasis model assessment of insulin resistance (HOMA‐IR) was calculated as the product of fasting serum insulin (μU/ml) and fasting plasma glucose (FPG, mmol/l) divided by 22.5.^26^ The estimated glomerular filtration rate (eGFR) was calculated using the abbreviated Modification of Diet in Renal Disease formula refitted to be more appropriate for the Chinese population: eGFR = 186 × [serum creatinine × 0.011]^−1.154^ × [age] ^−0.203^ × [0.742 if female] × 1.233.^27^

### 2.3 Definition of glycemic status

The glycemic status including normal glycemic regulation (NGR), prediabetes, and diabetes was defined based on the American Diabetes Association criteria.^28^ Specifically, NGR was defined by FPG < 5.6 mmol/l (100 mg/dl), 2‐h post‐load glucose (PG) < 7.8 mmol/l (140 mg/dl), and HbA1c < 5.7% in participants without previous diagnosis of diabetes. Prediabetes was defined by FPG between 5.6 and 6.9 mmol/l (100–125 mg/dl), 2‐h‐PG between 7.8 and 11.0 mmol/l (140–199 mg/dl), or HbA1c between 5.7% and 6.4% in participants without previous diagnosis of diabetes. Diabetes was defined by FPG ≥ 7.0 mmol/l (126 mg/dl), 2‐h‐PG ≥11.1 mmol/l (200 mg/dl), HbA1c ≥ 6.5%, or a self‐reported diagnosis of diabetes with or without current use of antidiabetic medications.

### 2.4 Measurement of ba‐PWV

Ba‐PWV was measured using Colin VP‐1000 (Model BP203RPE II, form ankle‐brachial index/PWV) by trained staff. After resting for at least 10 min, participants were asked to keep cuffs around their left and right arms and ankles, and the pulse wave was automatically detected at the same time on each side. Ba‐PWV was calculated by dividing the distances between arm and ankle by the transit time between the start of brachial and tibial pulse waves. The larger bilateral value of ba‐PWV was used in the current analysis.

### 2.5 Statistical analysis

Baseline characteristics of the participants are shown by glycemic status and sex. Continuous variables with normal distribution were presented as means (SDs). Skewed variables such as HbA1c and triglycerides were presented as medians (interquartile ranges) and logarithmically transformed for statistical tests. Categorical variables were presented as numbers (percentages). Comparisons were conducted between men and women in each stratum of glycemic status by Student's *t* tests for continuous variables and chi‐square tests for categorical variables. Restricted cubic splines were used to investigate the relationships between glucose levels, insulin levels, HOMA‐IR, and ba‐PWV levels with three knots at 5th, 50th, and 95th percentiles. Means and SDs of ba‐PWV were calculated and compared in men vs women with NGR, prediabetes, or diabetes, respectively, overall and stratified by age (40–59 vs ≥ 60 years). Smooth trajectories of ba‐PWV levels by age in men and women in strata of glycemic status were presented using generalized additive models to flexibly fit the possible trend of ba‐PWV with an increase in age. Unadjusted, age‐adjusted, and multivariable adjusted linear regression models were used to examine the association between sex and ba‐PWV levels in participants with NGR, prediabetes, or diabetes, overall and stratified by age. Covariates in the multivariable model included age, education level (high school and above, yes/no), current smoking (yes/no), current drinking (yes/no), physical activity (physically active/inactive), BMI, systolic blood pressure, triglycerides, LDL‐c, HDL‐c, and eGFR. Interaction terms were added to the models to examine the interaction effect of glycemic status on the association between sex and ba‐PWV, overall and stratified by age. Participants were also categorized into groups of with and without insulin resistance defined by HOMA‐IR ≥2.5.^29^ The association between sex and ba‐PWV levels in insulin resistance categories and potential modifications by age were examined in the unadjusted, age‐adjusted, and multivariable adjusted linear regression models. The statistical analysis was performed with R version 4.1.2 and a two‐sided *p* value of less than .05 was judged as having statistical significance.

## 3 RESULTS

**FIGURE 1:**
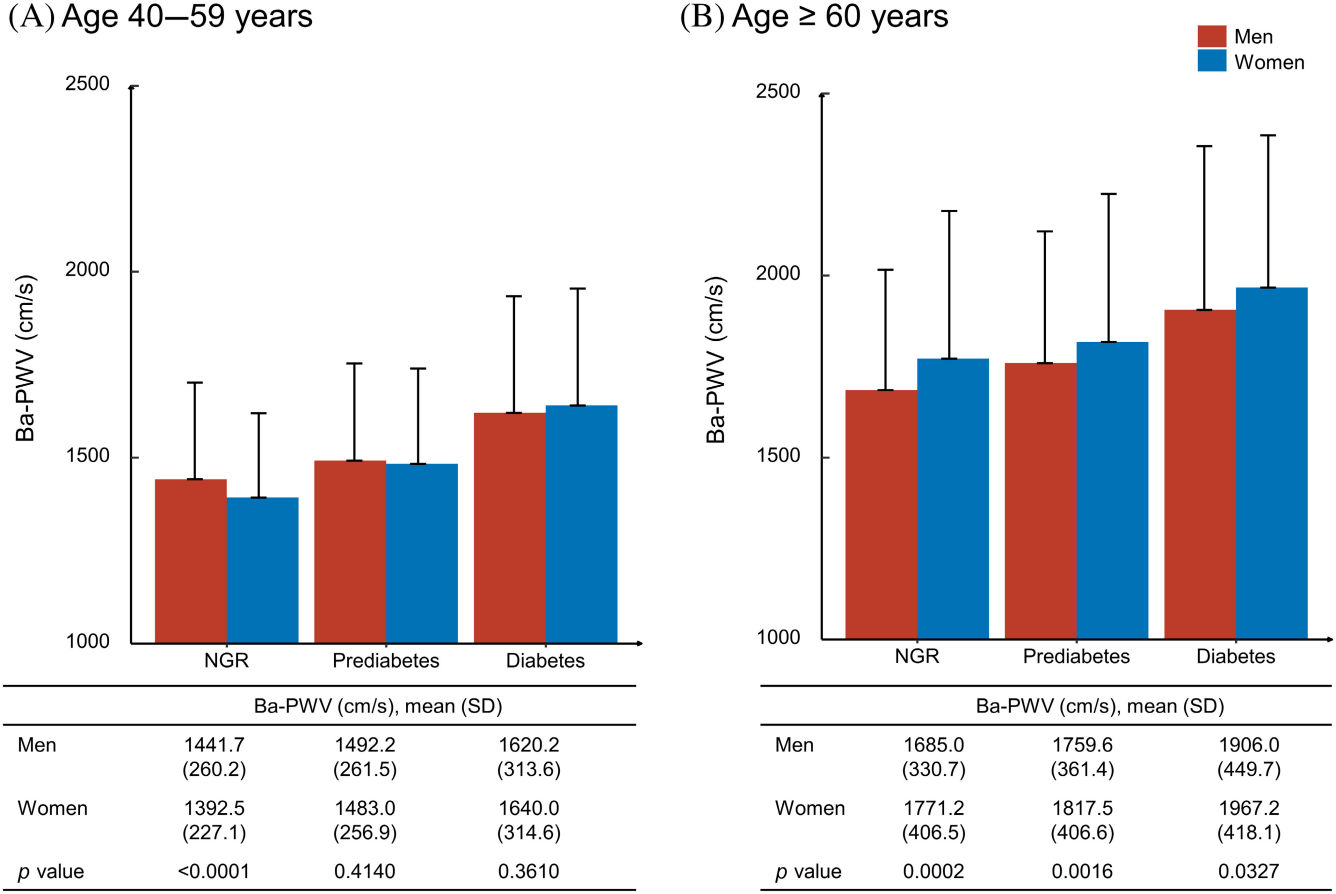
Means and SDs of ba‐PWV levels by sex and glycemic status in participants with age 40–59 years (A) and ≥60 years (B). ba‐PWV: brachial‐ankle pulse wave velocity; NGR, normal glycemic regulation.

As shown in Table 1, 1391 men and 2186 women with NGR, 1475 men and 2736 women with prediabetes, 761 men and 1069 women with diabetes were included in the current analysis. Overall, men were more likely to be educated, current smokers, and current drinkers and had lower levels of LDL‐C and HDL‐C compared with women in all glycemic strata. Furthermore, BMI, systolic blood pressure, triglycerides, ba‐PWV were higher in men than in women in NGR, comparable in both sexes in prediabetes, and lower in men than in women in diabetes. In participants aged 40–59 years, a similar trend to the overall study population can be found although not quite significant in the diabetes group. However, in participants aged ≥60 years, women had significantly higher levels of ba‐PWV than men in all glycemic strata (Figure 1).

**TABLE 1:** Baseline characteristics of participants stratified by glycemic status and sex

Characteristics	NGR		*p* value	Prediabetes		*p* value	Diabetes		*p* value
	Men	Women		Men	Women		Men	Women	
Number of participants	1391	2186	/	1475	2736	/	761	1069	/
Age (years)	56.5 (9.8)	55.1 (9.1)	<.001	59.6 (9.5)	58.9 (9.0)	.031	60.5 (9.7)	61.7 (8.9)	.007
High school education or above, *n* (%)	375 (27.1)	536 (24.6)	.107	334 (22.7)	481 (17.7)	<.001	211 (27.8)	152 (14.3)	<.001
Current smoking, *n* (%)	788 (57.4)	10 (0.5)	<.001	774 (53.1)	12 (0.5)	<.001	366 (48.7)	3 (0.3)	<.001
Current drinking, *n* (%)	348 (25.6)	14 (0.7)	<.001	395 (27.2)	27 (1.0)	<.001	178 (23.9)	6 (0.6)	<.001
Physically active, *n* (%)	187 (13.5)	318 (14.6)	.370	199 (13.5)	422 (15.5)	.100	112 (14.7)	181 (16.9)	.227
BMI (kg/m^2^)	24.7 (3.0)	24.1 (3.0)	<.001	25.4 (3.1)	25.3 (3.2)	.220	26.0 (3.2)	26.3 (3.6)	.077
Systolic BP (mmHg)	136.6 (18.2)	134.0 (19.5)	<.001	141.7 (18.2)	142.8 (20.1)	.106	146.4 (19.2)	150.4 (19.9)	<.001
Diastolic BP (mmHg)	83.5 (10.2)	80.1 (10.0)	<.001	85.1 (10.1)	82.5 (10.2)	<.001	85.7 (10.4)	82.8 (10.3)	<.001
FPG (mmol/l)	4.84 (0.40)	4.84 (0.38)	.883	5.36 (0.62)	5.27 (0.55)	<.001	7.80 (2.58)	7.24 (2.32)	<.001
2‐h PG (mmol/l)	5.55 (1.21)	5.88 (1.06)	<.001	7.17 (1.94)	7.56 (1.70)	<.001	15.20 (5.37)	14.77 (5.21)	.087
HbA1c (%)	5.4 [5.2, 5.5]	5.4 [5.2, 5.5]	.018	5.7 [5.6, 5.9]	5.7 [5.6, 5.9]	.271	6.7 [6.0, 7.8]	6.5 [6.0, 7.2]	<.001
Fasting insulin (μU/ml)	5.40 [3.50, 7.88]	6.33 [4.60, 8.79]	<.001	6.08 [4.00, 9.10]	7.60 [5.40, 10.80]	<.001	7.60 [4.80, 11.70]	9.80 [6.41, 14.02]	<.001
2‐h post‐load insulin (μU/ml)	28.4 [16.4, 45.5]	37.5 [24.5, 54.2]	<.001	40.8 [23.1, 69.0]	55.3 [35.1, 86.5]	<.001	42.7 [22.4, 72.4]	61.6 [35.6, 102.2]	<.001
HOMA‐IR	1.17 [0.74, 1.71]	1.37 [0.97, 1.90]	<.001	1.43 [0.95, 2.20]	1.77 [1.22, 2.56]	<.001	2.50 [1.52, 3.95]	2.97 [1.93, 4.52]	<.001
LDL‐C (mmol/l)	2.90 (0.72)	3.13 (0.85)	<.001	3.11 (0.80)	3.36 (0.88)	<.001	3.14 (0.89)	3.41 (0.93)	<.001
HDL‐C (mmol/l)	1.24 (0.30)	1.42 (0.31)	<.001	1.26 (0.31)	1.38 (0.32)	<.001	1.20 (0.31)	1.32 (0.30)	<.001
Triglycerides (mmol/l)	1.28 [0.91, 1.80]	1.18 [0.88, 1.64]	<.001	1.37 [0.97, 1.96]	1.44 [1.02, 2.03]	.012	1.61 [1.13, 2.38]	1.67 [1.21, 2.26]	.230
eGFR (ml/min/1.73 m^2^)	135.9 (24.3)	138.8 (24.7)	.001	133.8 (24.2)	136.6 (25.9)	.001	139.3 (32.0)	140.8 (31.2)	.322
ba‐PWV (cm/s)	1523.4 (308.0)	1489.0 (328.2)	.002	1617.9 (339.7)	1622.6 (367.0)	.682	1765.2 (413.8)	1820.6 (408.9)	.004

*Note*: Continuous variables are presented as means (SDs) or medians [interquartile ranges], and categorical variables are presented as absolute numbers (percentages).

Abbreviations: 2‐h PG, 2‐h post‐load glucose; ba‐PWV, brachial‐ankle pulse wave velocity; BMI, body mass index; BP, blood pressure; eGFR, estimated glomerular filtration; FPG, fasting plasma glucose; HbA1c, hemoglobin A1c; HDL‐C, high‐density lipoprotein cholesterol; HOMA‐IR, homeostasis model of insulin resistance; LDL‐C, low‐density lipoprotein cholesterol; NGR, normal glycemic regulation.

**FIGURE 2:**
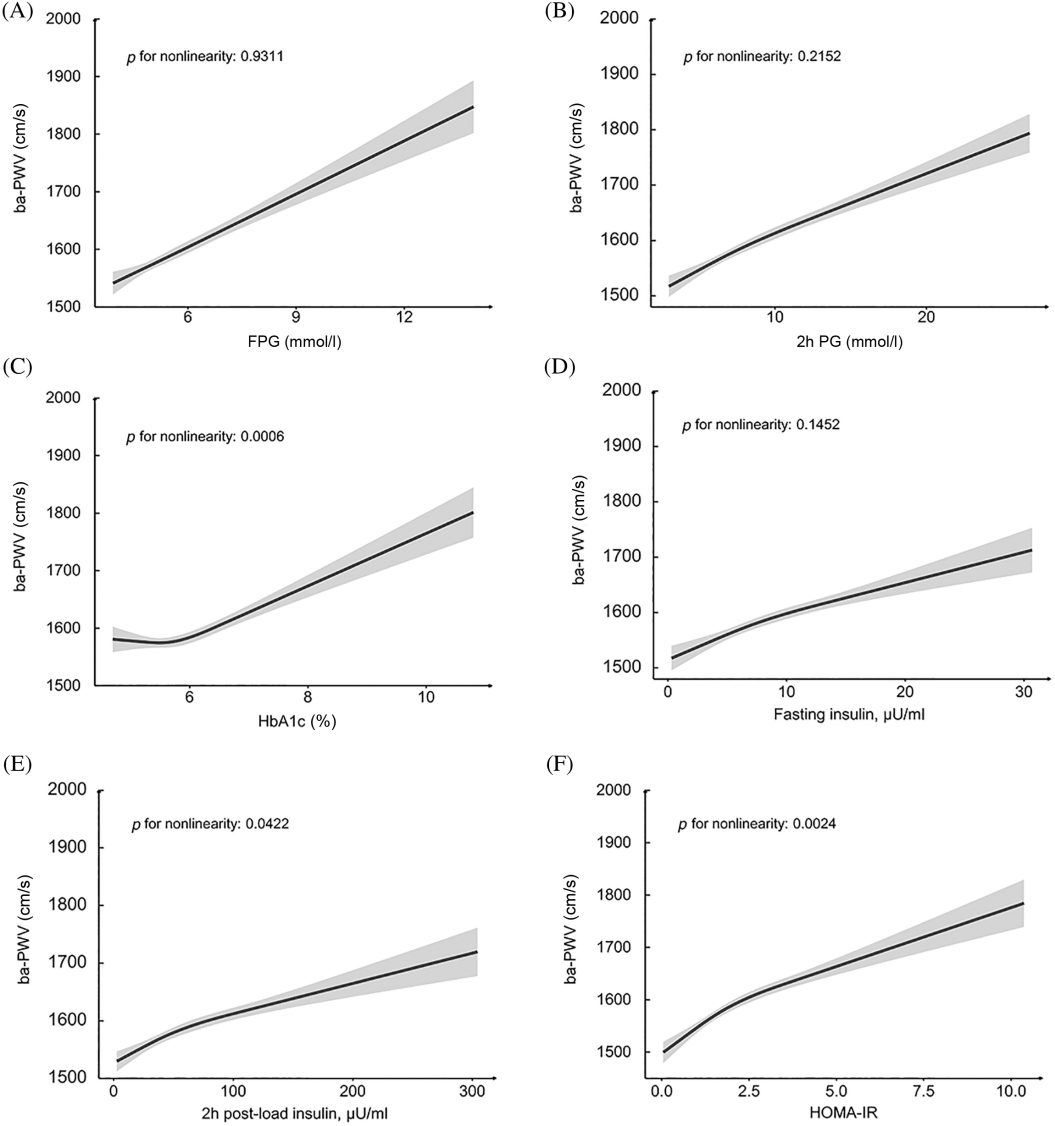
Associations between fasting plasma glucose (FPG) (A), 2‐h post‐load glucose (2‐h PG) (B), hemoglobin A1c (HbA1c) (C), fasting insulin (D), 2‐h post‐load insulin (E), homeostasis model of insulin resistance (HOMA‐IR) (F), and brachial‐ankle pulse wave velocity (ba‐PWV) levels. Shading areas indicate 95% confidence intervals. The model was adjusted for sex, age, education level, smoking and drinking status, physical activity, body mass index, systolic blood pressure, triglycerides, low‐density lipoprotein cholesterol, high‐density lipoprotein cholesterol, and estimated glomerular filtration rate.

**FIGURE 3:**
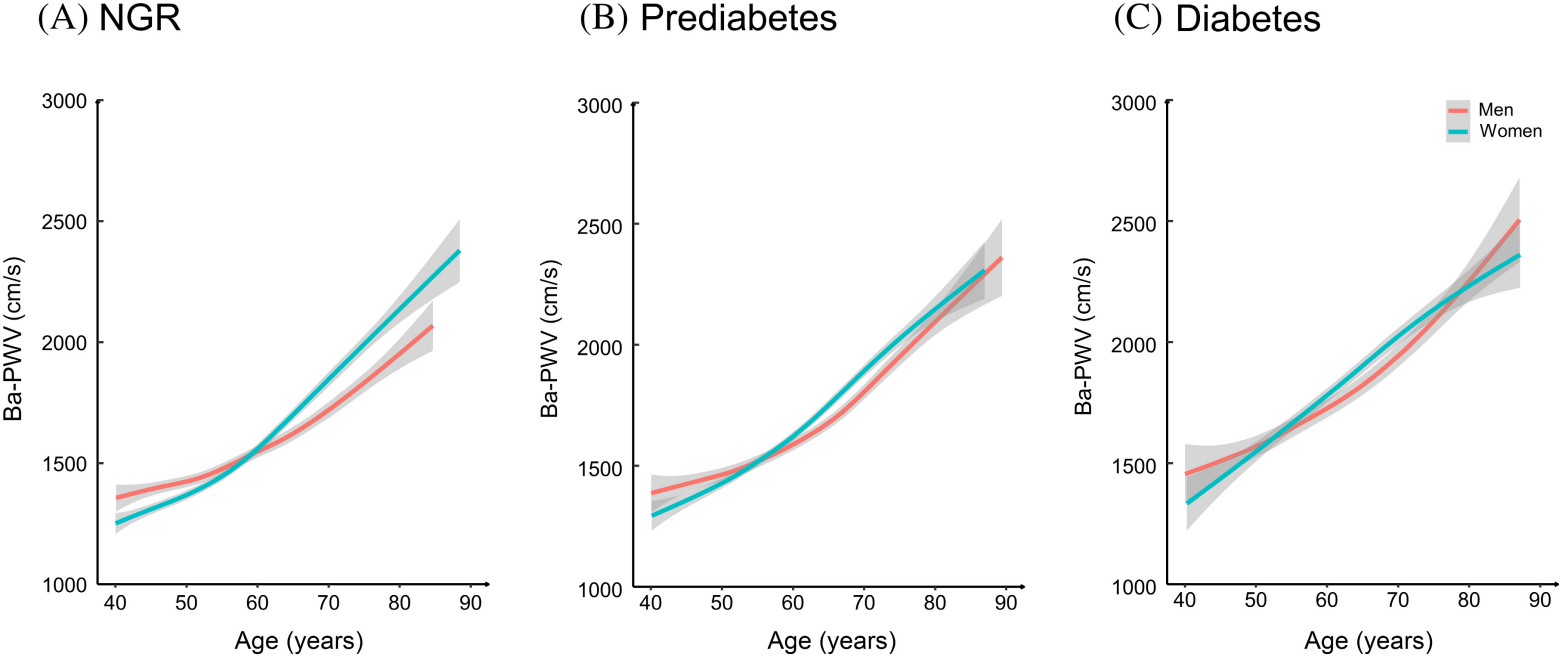
Smooth trajectories of ba‐PWV levels by age and sex in participants with NGR (A), prediabetes (B), or diabetes (C). Solid lines are means and gray areas indicate 95% confidence intervals. ba‐PWV: brachial‐ankle pulse wave velocity; NGR: normal glycemic regulation.

Associations between glucose levels, insulin levels, HOMA‐IR, and ba‐PWV are shown in Figure 2. Monotonically increasing linear associations were found between FPG, 2‐h PG, insulin levels, and ba‐PWV levels. The smooth trajectories of mean ba‐PWV by age and sex in different strata of glycemic status are shown in Figure 3. In participants with NGR, levels of ba‐PWV were higher in men than in women until age 60 years, after which levels of ba‐PWV were significantly higher in women than in men. In participants with prediabetes or diabetes, although the pattern seemed similar, levels of ba‐PWV were close between men and women, especially in those aged ≥60 years.

**FIGURE 4:**
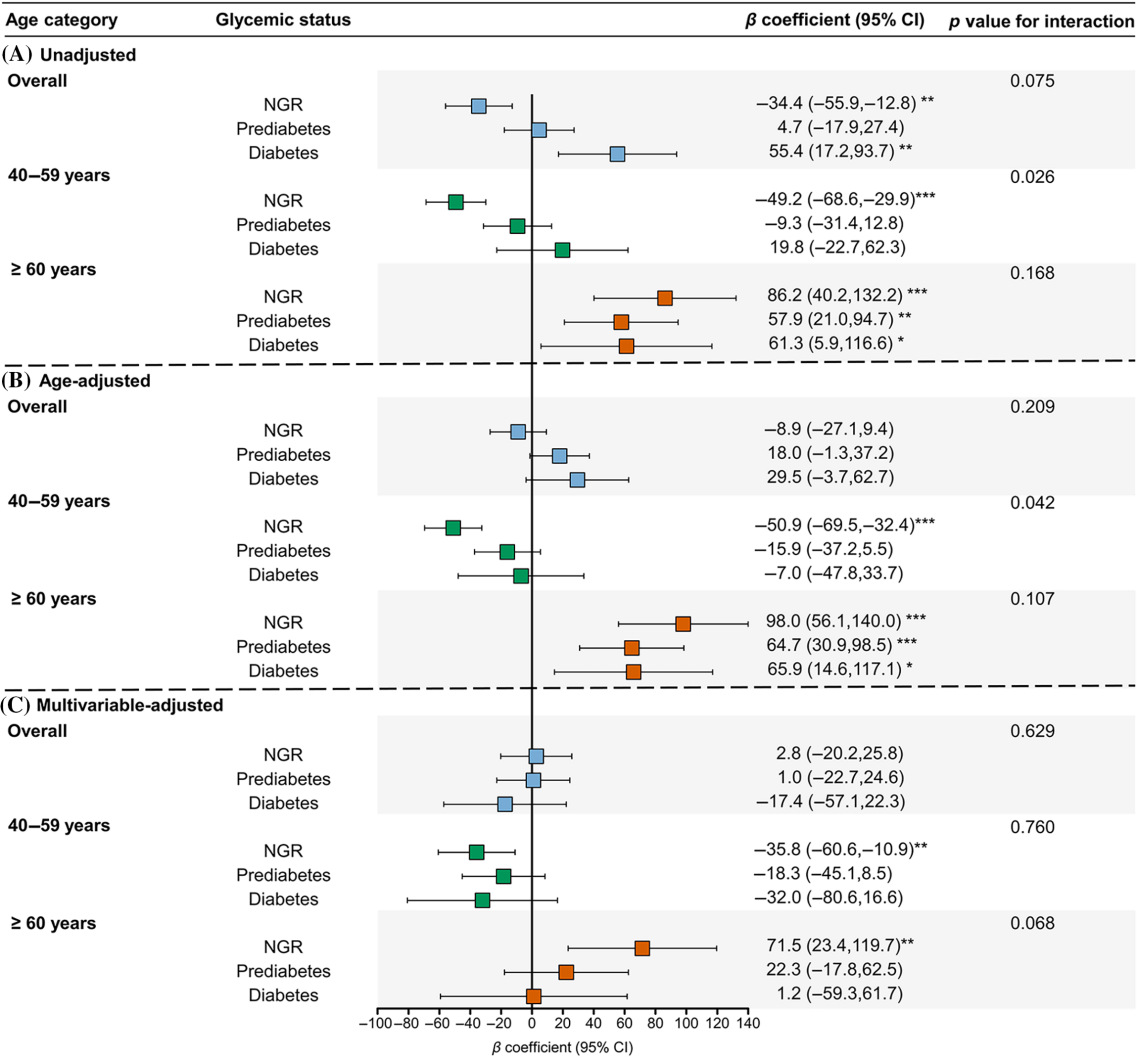
Unadjusted (A), age‐adjusted (B), and multivariable‐adjusted (C) associations between sex (women vs men) and ba‐PWV levels in participants with NGR, prediabetes, or diabetes in the overall study population and in age groups. The multivariable‐adjusted model was adjusted for age, education level, smoking and drinking status, physical activity, body‐mass index, systolic blood pressure, triglycerides, low‐density lipoprotein cholesterol, high‐density lipoprotein cholesterol, and estimated glomerular filtration rate. ba‐PWV, brachial‐ankle pulse wave velocity; CI, confidence interval; NGR: normal glycemic regulation. *: *p* < .05, **: *p* < .01, ***: *p* < .001

**FIGURE 5:**
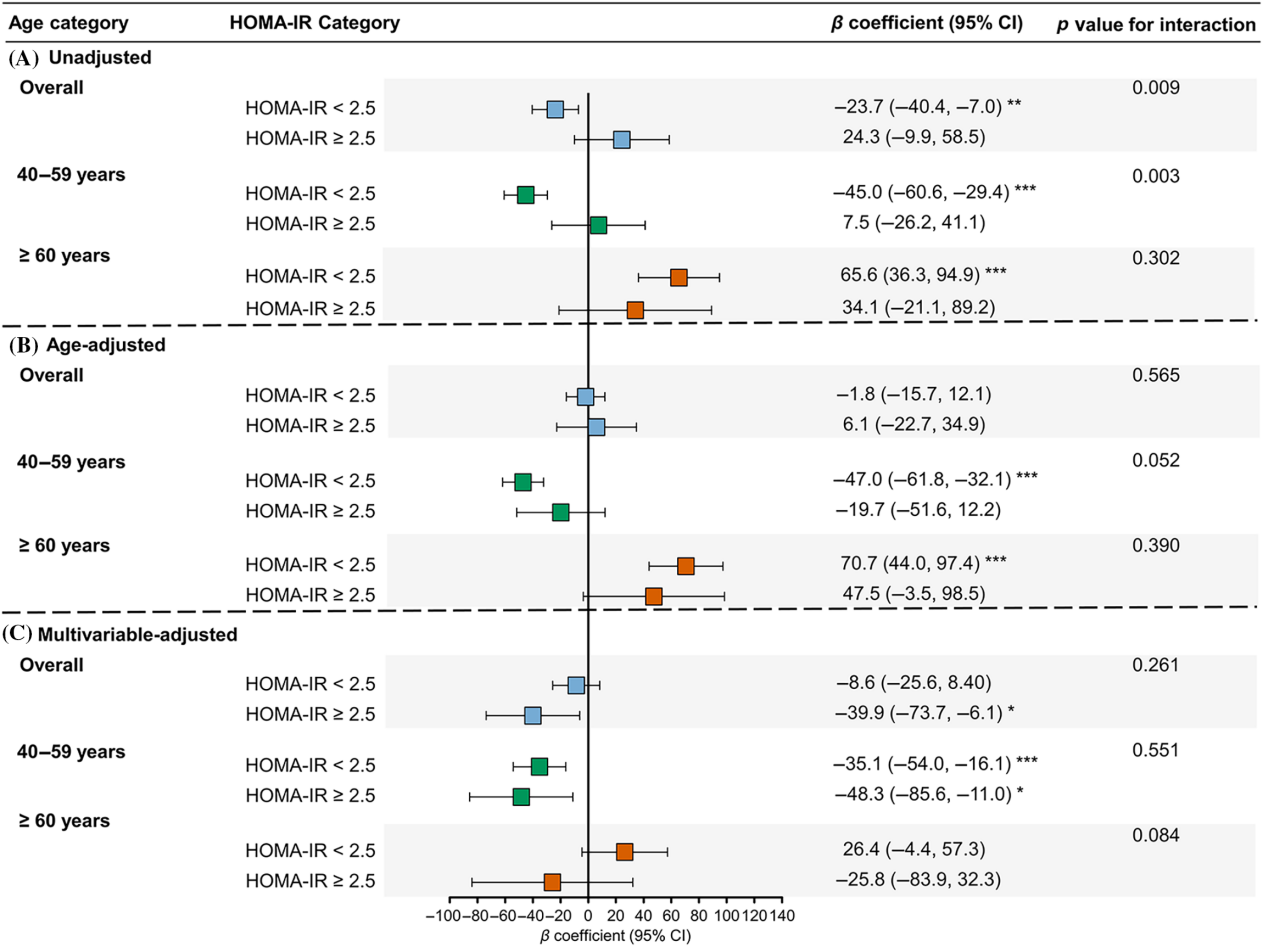
Unadjusted (A), age‐adjusted (B), and multivariable‐adjusted (C) associations between sex (women vs men) and ba‐PWV levels in HOMA‐IR categories in the overall study population and in age groups. The multivariable‐adjusted model was adjusted for age, education level, smoking and drinking status, physical activity, body mass index, systolic blood pressure, triglycerides, low‐density lipoprotein cholesterol, high‐density lipoprotein cholesterol, and estimated glomerular filtration rate. ba‐PWV: brachial‐ankle pulse wave velocity; CI, confidence interval; HOMA‐IR, homeostasis model of insulin resistance. *: *p* < .05, **: *p* < .01, ***: *p* < .001

Using men as the reference, sex difference in association with ba‐PWV levels in participants with different glycemic status was examined in Figure 4. In the unadjusted model, women were associated with lower ba‐PWV in the NGR group (*β* coefficient = −34.4; 95% confidence interval [CI] = −55.9, −12.8), comparable ba‐PWV in the prediabetes group (*β* = 4.7, 95% CI = −17.9, 27.4), and higher ba‐PWV in the diabetes group (*β* = 55.4, 95% CI = 17.2, 93.7) (Figure 4A). However, adjustment for age changed the pattern (Figure 4B). Further adjustment for other confounders revealed that women were associated with lower ba‐PWV levels in generally all glycemic strata in participants aged 40–59 years. However, in participants aged ≥60 years, women were associated with significantly higher ba‐PWV levels in the group of NGR after multivariable adjustment and this sex difference was attenuated in prediabetes and diabetes with borderline significant interaction between sex and glycemic status (*p* for interaction = .068) (Figure 4C). Similar results were found for HOMA‐IR categories (Figure 5).

## 4 DISCUSSION

In this large community‐based population study, we found that the sex difference in arterial stiffness evaluated by ba‐PWV in adults with different glycemic status was highly dependent on age. In adults aged 40–59 years, the female sex was mostly “protective” regardless of the glycemic status. However, in adults aged ≥60 years, the female sex was identified as a “risk” factor of arterial stiffness in NGR, whereas this sex difference tended to diminish with the deterioration of glycemic regulation. These findings provide new evidence for prioritizing preventive treatment against adverse cardiovascular events in men and women with different glycemic status.

Few studies have examined specifically the sex difference in atherosclerosis across the glucose tolerance continuum. In the general population, men were reported to have a higher burden of subclinical atherosclerosis such as the carotid or femoral atherosclerotic plaques,^30^ coronary artery calcification (CAC),^31^ or elevated ba‐PWV.^32^ Evidence of sex difference in adults with glucose dysregulation can be found only from studies that reported findings in men and women subgroups. For example, in adults with prediabetes, no heterogeneity of associations between prediabetes and atherosclerosis indexes such as CAC, carotid wall thickness, or urinary albuminuria were observed in men and in women, indicating a lack of sex difference.^33^ Women seemed to have a higher risk of carotid atherosclerosis when having diabetes, as compared with men.^34^ Results from the current study before adjustment for age were generally similar with previous reports, showing that ba‐PWV, an indicator of arterial stiffness, was higher in men than in women in NGR, comparable in both sexes in prediabetes, and lower in men than in women in diabetes. However, adjustment for age or stratification by age groups revealed different findings.

Aging is recognized as a key player in the progression of arterial stiffness and imposes distinctive influence on different parts of the arterial tree.^35^, ^36^ It is putative that the development of arterial stiffness during aging might vary between sexes and findings from previous studies are controversial. Some studies reported that arterial stiffness developed similarly with aging in both sexes,^21^ and others demonstrated that aging affected arterial stiffness differently in men vs women and seemed to be more detrimental in women.^19^, ^20^, ^37^ Findings from the current study revealed that ba‐PWV was higher in men vs women until approximately 60 years of age, after which ba‐PWV was lower in men vs women. Therefore, aging has to be considered when sex difference in arterial stiffness was examined. The current study has assessed the relationships between sex, glycemic status, and arterial stiffness with stratification on age <60 and ≥60 years, providing comprehensive evidence regarding sex disparities in disease risks.

After multivariable adjustment, lower levels of ba‐PWV were always found in women vs men regardless of glycemic status in participants aged 40–59 years (*p* value for interaction = .760). After age 60, women were associated with significantly higher ba‐PWV values in participants with NGR. The sex difference was attenuated in participants with prediabetes and was diminished in participants with diabetes. In this age group, the *p* value for interaction between sex and glycemic status was marginally significant (.068), suggesting that there might be differences regarding sex disparities among participants with varied glycemic status. Previous studies have demonstrated the potential mechanisms for the augmentation of the age‐related increase in arterial stiffness in women and this could probably in part be associated with estrogen deficiency whereas at least 6 years after menopause were needed to achieve the significant risk of higher ba‐PWV.^19^, ^38^ The beneficial effects of estrogen therapy to increase arterial compliance and decrease PWV were also found.^39^ Likewise, androgen might also play a role in the change of sex‐specific associations with arterial stiffness in different glycemic regulation status probably by regulation of the gut microbiome affecting glucose metabolism.^38^, ^40^ In addition to sexual hormones, oxidative stress‐related inflammation might be another contributor to the accelerated deterioration of arterial stiffness across the menopausal transition period.^41^ Moreover, increased levels of cardiovascular risk factors such as BMI, blood pressure, etc. after menopause in women may also count.^20^ However, mechanisms that contribute to the sex difference in arterial stiffness in different glycemic status modified by aging still warrant further investigation.

The strengths of the current study included the comprehensive evaluation of glycemic status based on OGTT and HbA1c, arterial stiffness evaluated by ba‐PWV as an early indicator of atherosclerosis, taking aging into account during the analysis. The current study has several limitations. First, this is a cross‐sectional analysis. However, sex, which is the exposure variable, is a biological attribute determined before birth. Therefore, the temporality of associations in the current study can be easily settled. Second, we did not use the carotid‐femoral pulse wave velocity (cf‐PWV), which is regarded as the gold standard to assess arterial stiffness. However, ba‐PWV is less operator dependent than cf‐PWV and appropriate for large‐scale epidemiological studies. Besides, ba‐PWV correlates well with cf‐PWV.^42^ Third, levels of sexual hormones such as estrogen and androgen were not available. In addition, although many confounders were adjusted in the multivariable model, residual confounding cannot be avoided. Fourth, the determination of glucose levels was conducted only once and misclassification of glycemic status may exist. In addition, associations between prediabetes and arterial stiffness may depend on the glucose parameter that is elevated,^43^, ^44^, ^45^ but types of prediabetes (ie, impaired fasting glucose, impaired glucose tolerance, or elevated HbA1c) were not specified owing to a limited number of cases. Because insulin resistance and low‐grade inflammation, which often coexist with prediabetes, are risk factors leading to atherosclerosis,^46^, ^47^, ^48^ analyses in participants with and without insulin resistance provided additional insights. Fifth, although the overall study sample is large, the number of participants can be limited in certain groups of glycemic status and age. Finally, the current findings were from middle‐aged and elderly community adults living in Shanghai, which has limited generalizability to other populations.

In conclusion, findings from the current study unraveled that sex differences in arterial stiffness changed with aging among different glycemic status. Women might have lower risks of arterial stiffness than men regardless of glycemic status when they are with age 40–59 years. After age 60, women might have higher risks of arterial stiffness than men when glucose levels are normal but sex differences were attenuated when glucose levels are increased. The sex‐specific risk in atherosclerosis with glucose metabolism and aging warrants further investigation.

## AUTHOR CONTRIBUTIONS

Xiaoyun Zhang and Qianqian Yang made substantial contributions to the conceptualization, formal analysis, methodology, and original draft writing of the work. Ruizhi Zheng, Zhiyun Zhao, Mian Li, Tiange Wang, Min Xu, Jieli Lu, Shuangyuan Wang, and Hong Lin made substantial contributions to the data curation, investigation, review, and revision of the original work. Weiqing Wang, Guang Ning, and Yufang Bi made substantial contributions to the funding acquisition, investigation, resources, supervision, review, and revision of the original work. Yu Xu and Yuhong Chen made substantial contributions to the conceptualization, data curation, methodology, validation, review, and revision of the original work. All authors critically revised the manuscript, approved the final version of the manuscript, and agreed to be held responsible for all aspects of the work.

## DISCLOSURE

The authors declare no conflicts of interest.

## Data Availability

The data underlying this article cannot be shared publicly due to the privacy protection of individuals that participated in the study considering the ethics. The data will be shared upon reasonable request to the corresponding author.
